# Multivariate genome-wide covariance analyses of literacy, language and working memory skills reveal distinct etiologies

**DOI:** 10.1038/s41539-021-00101-y

**Published:** 2021-08-19

**Authors:** Chin Yang Shapland, Ellen Verhoef, George Davey Smith, Simon E. Fisher, Brad Verhulst, Philip S. Dale, Beate St Pourcain

**Affiliations:** 1 MRC Integrative Epidemiology Unit, University of Bristol, Bristol, UK; 2 Population Health Sciences, University of Bristol, Bristol, UK; 3 Language and Genetics Department, Max Planck Institute for Psycholinguistics, Nijmegen, The Netherlands; 4 International Max Planck Research School for Language Sciences, Nijmegen, The Netherlands; 5 Donders Institute for Brain, Cognition and Behaviour, Radboud University, Nijmegen, The Netherlands; 6 Texas A&M University, College Station, TX USA; 7 Speech & Hearing Sciences, University of New Mexico, Albuquerque, NM USA

**Keywords:** Communication, Human behaviour

## Abstract

Several abilities outside literacy proper are associated with reading and spelling, both phenotypically and genetically, though our knowledge of multivariate genomic covariance structures is incomplete. Here, we introduce structural models describing genetic and residual influences between traits to study multivariate links across measures of literacy, phonological awareness, oral language, and phonological working memory (PWM) in unrelated UK youth (8–13 years, *N* = 6453). We find that all phenotypes share a large proportion of underlying genetic variation, although especially oral language and PWM reveal substantial differences in their genetic variance composition with substantial trait-specific genetic influences. Multivariate genetic and residual trait covariance showed concordant patterns, except for marked differences between oral language and literacy/phonological awareness, where strong genetic links contrasted near-zero residual overlap. These findings suggest differences in etiological mechanisms, acting beyond a pleiotropic set of genetic variants, and implicate variation in trait modifiability even among phenotypes that have high genetic correlations.

## Introduction

Within most Indo-European languages, including English, an alphabetic writing system maps sequences of symbols to the sounds and meaning of words^1^. Thus, symbols or graphemes (letters or groups of letters) represent individual sounds (phonemes). This correspondence of spoken language to printed words, known as the alphabetic principle, is, in turn, the basis of phonological decoding skills, which enable the interpretation of texts through phonetic transformation^2^. As a consequence, close and reciprocal interrelationships manifest between phonological decoding, letter knowledge, and phonological awareness, the ability to dissect and transform words according to their phonological structure^3^. As links emerge and can be used in real-time to identify printed words, children apply this knowledge to develop reading and spelling skills^1^. Fully developed reading comprehension skills also include the ability to identify and integrate word meanings along with contextual and world knowledge to construct the meanings of sentences and larger discourse structures^4^. Once mastered, reading has a profound impact on the acquisition of knowledge, including print exposure^5^, and, ultimately, on final educational attainment^6^.

Based on this framework, the “Simple View of Reading”^4,7^ proposed that reading comprehension is the product of two independent skill sets, “decoding” and “language comprehension”, where the latter specifically refers to listening comprehension^7^, i.e. the ability to interpret the meaning of words in grammatical structures (sentences) presented orally^7^. In younger children, reading comprehension is thought to be constrained primarily by decoding abilities, whereas in older children, with a high level of decoding ability, reading comprehension is mainly a function of language comprehension^8^. Other theoretical approaches, such as the Reading Systems Framework^9^ implicate additional general cognitive resources in reading processes, such as phonological working memory (PWM)^10^, which is typically assessed by non-word repetition tasks^11^. This task requires a purely sound-based division of non-words along with retention and later reproduction of the sound sequence^10^. Like phonological awareness, PWM contributes substantially to decoding abilities. In addition, children with language and reading problems have lower non-word repetition accuracy that may affect receptive vocabulary development^12^.

Recent meta-analytic structural equation modeling studies have reported strong to moderate phenotypic interrelations between language, literacy, and related traits, such as working memory, spanning childhood and adolescence^13^. A considerable part of these relationships can be attributed to shared genetic factors as shown by twin research^14–22^ and by studies of unrelated individuals using genome-wide genotyping information^23^. These findings add to the widely established evidence that these abilities have moderate to strong heritability from mid-childhood onwards^14,18–26^. Such traits include reading (decoding, fluency, and comprehension)^18,19,21–23^, spelling^18,22,23^, phonological awareness^20,21,23^, language comprehension^14,16,23^, and PWM (assessed via non-word repetition tests^11^)^23^. Moreover, some developmental genetic stability has been observed for academic measures of reading achievement^24^ and both reading comprehension and fluency, a composite of decoding accuracy and rate^14^, despite children’s increasing proficiency in decoding abilities with age^8^, accompanied by developmental genetic stability in oral language performance^14^.

This wide-spread evidence of genetic pleiotropy is consistent with the existence of “generalist genes” that contribute to shared aspects of cognitive functioning, such as those involved in literacy, language, working memory and related skills, affecting in particular learning abilities^27^. As hypothesized by a recent “multiple-deficit theory” model, “generalist genes” may play a role in increasing liability to developmental disorders, including reading problems in dyslexia^28,29^. This is supported by observational research demonstrating that cumulative risk is the best predictor for poor language and reading^30^. However, we lack a comprehensive map of genetic covariance structures, which include both these broad connections as well as unique genetic relationships.

For example, strong genetic links have been identified between oral language and reading comprehension^14,16^, while genetic overlap between oral language and word reading fluency has been only moderate^14–16^. Genetic differences may, thus, partially distinguish meaning-based (i.e. comprehension-related) versus code-based (i.e. decoding-related) abilities^14–16^, consistent with the two etiologically distinct core factors proposed by the “Simple View of Reading”, potentially shaping detectable overarching multivariate genetic covariance patterns across domains. Even within a domain, such as literacy, reading fluency measures may subtly vary in the level of comprehension- and decoding-related aspects required and, consequently, differ in trait interrelationships. However, investigations of multiple measures of reading fluency, allowing inferences beyond measurement-specific limitations, are still outstanding, and shared genetic links with PWM capturing underlying cognitive resources during mid-childhood and early adolescence have not yet been characterized.

Importantly, the current understanding of multivariate genetic covariance across these phenotypic domains has been largely based on twin analyses^14,17–23^. While twin studies offer many advantages, notably the ability to disentangle genetic from shared environmental and nonshared environmental factors^31^, they have also been criticized for their reliance on several assumptions. This includes, for example, the equal environment assumption for mono- and dizygotic twins, in particular the equal treatment of twins, the correct classification of zygosity and the representativeness of twin studies for the general population^31^. Investigations of multivariate genetic structures with independent approaches, utilizing general population-based samples, have, so far, been missing.

This study aims to evaluate the genetic relation of reading skills to abilities that are outside of reading proper but are nonetheless literacy-related. Reading is a complex trait that builds upon numerous, interrelated skills that each have a myriad genetic and environmental causes. As people learn to read they must integrate these disparate, complex abilities into a fluent, nearly automatic skill. Understanding how these interrelated skills culminate in an individual’s ability to read provides a framework for modifying the way we teach students to read and ultimately allow us to enhance the effectiveness of the learning process.

Firstly, we model the multivariate genetic covariance across four phenotypic domains: literacy (assessed by reading fluency and spelling measures), phonological awareness (phonemic awareness), oral language (listening comprehension), and PWM (non-word repetition).

Secondly, by modeling genetic data from unrelated individuals, we use independent methods to confirm and augment knowledge of multivariate genetic architectures identified in twin studies. Specifically, we bypass twin research-related criticisms by investigating multivariate genetic trait covariance in unrelated individuals with genome-wide single nucleotide polymorphism (SNP) information^32^, studying youth from The Avon Longitudinal Study of Parents and Children (ALSPAC) birth cohort^33,34^. Here, we fit structural models to genetic relationship-matrices (GRMs), derived from genome-wide markers, using structural equation modeling (Genetic-relationship-matrix structural equation modeling, GSEM)^32^. Specifically, GSEM adapts multivariate twin models^35^ to make them applicable to GRM-based analyses, as originally proposed for genomic data using uni- and bivariate genomic-relatedness-based restricted maximum-likelihood (GREML)^36,37^. Like GREML^36^, GSEM dissects phenotypic variation into additive genetic variance (A) and residual variance (E). However, beyond estimating SNP-based heritability estimates (*SNP-h*^*2*^) and genetic correlations, GSEM models aim to identify underlying genetic factor structures. In addition to fitting independent pathway and Cholesky decomposition model, we also implement a novel combined Independent Pathway/Cholesky (IPC) model, enhancing the interpretability of genetic factor structures without a loss of model fit in residual factor structures. This approach enables us ultimately to compare genetic and residual covariance between traits to illuminate similarities and differences in etiological relationships between literacy (reading fluency, spelling), phonological awareness, oral language, and PWM.

## Results

### Univariate and bivariate genetic variance analyses

Variability in reading fluency (non-word-reading speed and accuracy, word reading speed and accuracy, passage reading speed and accuracy), spelling, phonemic awareness, listening comprehension, and non-word repetition during mid-childhood and adolescence (Table 1) was moderately heritable, confirming previous reports^23,38^. Using Genome-wide Complex Trait Analysis (GCTA) software, GREML-based SNP-based heritability (GCTA *SNP-h*^*2*^) estimates for reading fluency, spelling accuracy, phonemic awareness, listening comprehension, and non-word repetition ranged between 30% (SE = 0.06) and 50% (SE = 0.07) when assessed in unrelated children and adolescents from the general population, irrespective of phenotypic transformation (Table 1 and Supplementary Table 1). All scores were phenotypically (Supplementary Table 2) and genetically (Supplementary Tables 3 and 4) correlated (Supplementary Fig. 1) ranging between 0.2–0.8 and 0.4–0.98, respectively.

**Table 1:** Description of measures.

Measure	Mean age (SE) in years	%Males	*N*	GCTA *SNP-h*^*2*^ (SE)
P reading a 9 (NARA)	9.88 (0.32)	49.4	5048	0.50 (0.07)
P reading s 9 (NARA)	9.88 (0.32)	49.3	5037	0.45 (0.07)
W reading a 9 (NBO)	9.87 (0.32)	49.6	5574	0.46 (0.06)
W reading s 13 (TOWRE)	13.83 (0.20)	48.5	4131	0.40 (0.09)
NW reading a 9 (NBO)	9.87 (0.32)	49.5	5569	0.32 (0.06)
NW reading s 13 (TOWRE)	13.83 (0.20)	48.4	4121	0.38 (0.09)
Spelling a 7 (NB)	7.53 (0.31)	50.5	5637	0.32 (0.06)
Spelling a 9 (NB)	9.87 (0.32)	49.5	5564	0.38 (0.06)
Phon aware 7 (AAT)	7.53 (0.31)	50.9	5749	0.39 (0.06)
Listening compreh 8 (WOLD)	8.63 (0.30)	50.1	5324	0.32 (0.07)
NW repetition 8 (CNRep)	8.63 (0.30)	50.1	5315	0.32 (0.07)

SNP heritability (*SNP-h*^*2*^) was estimated with Genetic-relationship-matrix Restricted Maximum Likelihood (GREML) analysis using Genome-wide Complex Trait Analysis (GCTA) software. *SNP-h*^*2*^ are based on rank-transformed residuals.

Measures: P reading a 9 (NARA), passage reading accuracy (NARA II^54^); P reading s 9 (NARA), passage reading speed (NARA II^54^); W reading a 9 (NBO), word reading accuracy (ALSPAC-specific: NBO^52^); W reading s 13 (TOWRE), word reading speed (TOWRE^56^); NW reading a 9 (NBO), non-word reading accuracy (ALSPAC-specific: NBO^52^); NW reading s 13 (TOWRE), non-word reading speed (TOWRE^56^); Spelling a 7 (NB), spelling accuracy (ALSPAC-specific: NB^23^); Spelling a 9 (NB), spelling accuracy (ALSPAC-specific: NB^23^); Phon aware 7 (AAT), phonemic awareness (AAT^57^); Listening compreh 8 (WOLD), listening comprehension (WOLD^59^); NW repetition 8 (CNRep), and non-word repetition (CNRep^11^).

*AAT* Auditory Analysis Test, *ALSPAC* Avon Longitudinal study of Parents and Children, *CNRep* Children’s Test of Nonword Repetition, *N* number of participants with phenotype data and genetic data, *NARA II* The Neale Analysis of Reading Ability-Second Revised British Edition, *NBO* ALSPAC-specific assessment developed by Nunes, Bryant and Olson, *NB* ALSPAC-specific assessment developed by Nunes and Bryant, *TOWRE* Test Of Word Reading Efficiency, *PWM* Phonological working memory, *WOLD* Wechsler Objective Language Dimensions, *SE* standard error.

### Modeling strategy for multivariate analyses

Using a series of structural equation models, we describe the multivariate polygenic covariance of reading fluency, spelling, phonemic awareness, listening comprehension and non-word repetition abilities. Due to the computational burden of multivariate genetic variance analyses, we were not able to fit one large combined model of all 11 measures in this study. Instead, we adopted a two-stage approach: First, we fit multiple smaller-scale multivariate models focussed on literacy measures only with the aim of identifying proxy measures that sufficiently capture the observed genetic structures of reading fluency (6 measures) and spelling (2 measures), which were assessed with multiple psychological instruments (Stage 1, Supplementary Table 5). Second, we fitted multivariate genetic models across traits including the proxy measures of reading fluency and spelling (identified from Stage 1) as well as phonemic awareness, listening comprehension and non-word repetition (Stage 2, Supplementary Table 5).

For each fitted multivariate model during either stage (except for spelling), three GSEM submodels were examined: (i) a saturated model (Cholesky decomposition), (ii) an independent pathway model, and (iii) a hybrid IPC model, involving an independent pathway model for the genetic covariance structure and a Cholesky decomposition model for the residual covariance structure. The model fit was compared using Akaike information criterion (AIC) and Bayesian information criterion (BIC) fit indices and likelihood ratio tests (LRT)^39^. The two spelling measures (Stage 1) were fitted with a Cholesky decomposition model.

### Single-domain structural models of reading fluency

**Fig. 1:**
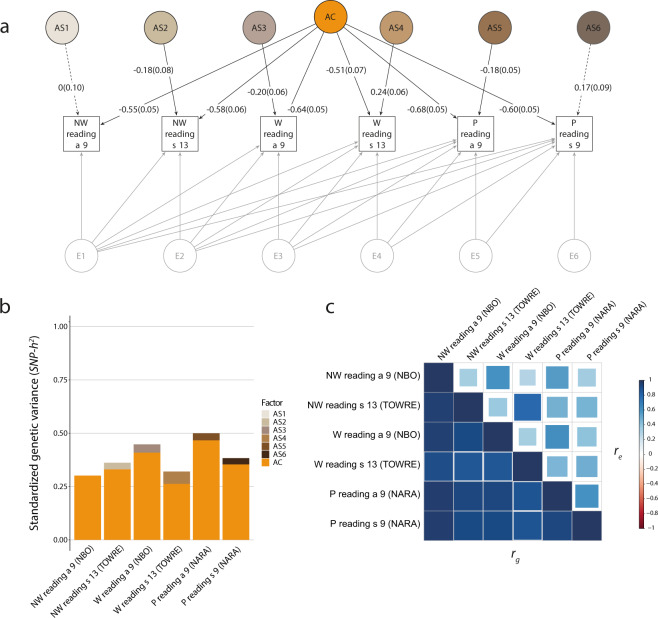
Single-domain structural model of reading fluency. Genetic-relationship matrix structural equation modeling (GSEM) of six reading fluency measures (*N* = 5866). The path diagram (**a**) depicts the genetic factors of the best-fitting model (IPC model) describing variation in non-word reading accuracy at age 9 (NW reading a 9, NBO), non-word reading speed at age 13 (NW reading s 13, TOWRE), word reading accuracy at age 9 (W reading a 9, NBO), word reading speed at age 13 (W reading s 13, TOWRE), passage reading accuracy at age 9 (P reading a 9, NARA), and passage reading speed at age 9 (P reading s 9, NARA). The phenotypic variance was dissected into common (AC) and specific (AS1-AS6) genetic factors, according to an Independent Pathway model, as well as residual factors, based on a Cholesky decomposition (E1-E6; Factor loadings are not shown, Supplementary Table 6, and Supplementary Fig. 3). Observed measures are represented by squares and factors by circles. Single-headed arrows (paths) define relationships between variables. Dotted and solid paths represent factor loadings with *p* > 0.05 and *p* ≤ 0.05 respectively. The variance of latent variables is constrained to unit variance; this is omitted from the diagram to improve clarity. The variance plot (**b**) depicts the standardized genetic variance components for the model in **a**. The correlogram (**c**) shows genetic (*r*_g_, lower triangle) and residual (*r*_e_, upper triangle) correlations for the model in **a**. Point estimates and their SEs are reported in Supplementary Table 9. All measures were rank-transformed. IPC model – Combined Independent Pathway/Cholesky model.

We started the process of proxy measure identification (Stage 1, Supplementary Table 5) by structurally modeling the six reading fluency measures with the aim to identify the proxy measure of reading fluency: non-word reading accuracy and speed, word reading speed and accuracy, and passage reading accuracy and speed, all ascertained in ALSPAC participants between the ages of 9–13 years (Table 1). Following BIC (the more stringent criterion), the most parsimonious model describing the data was the IPC model (Fig. 1, Table 2, and Supplementary Tables 6–9). The model identified evidence for a shared genetic factor (A) across the six reading fluency measures (Fig. 1), in addition to specific genetic factors for some of the measures.

**Table 2:** Single-domain GSEM model fit comparisons of reading fluency.

Model	LL	-2LL	*k*	AIC	BIC	Δ*χ*^2^ to Cholesky	Δ*df* to Cholesky	*p* value
Cholesky	−4488.71	8977.41	42	9061.41	9341.84	–	–	–
IPC	−4504.50	9009.00	33	9075.00	**9295.34**	31.59	9	2.3 × 10^−4^
IP	−4656.96	9313.91	24	9361.91	9522.16	336.50	18	<10^−10^

5866 participants had phenotype data of this domain and genetic data. The models were compared with likelihood ratio tests, AIC and BIC. The model with the lowest BIC values is shown in bold and its path diagram is shown in Fig. 1. All analyses are based rank-transformed residualized scores.

*AIC* Akaike information criterion, *BIC* Bayesian information criterion, *Cholesky* Cholesky decomposition (saturated) model, *GSEM* genetic-relationship matrix structural equation modeling, *IPC* joint independent pathway (genetic part)/Cholesky (residual part) model, *IP* independent pathway model, *LL* log-likelihood, *k* number of parameters.

For the shared genetic factor (Fig. 1a, b), the strongest factor loading was estimated for passage reading accuracy, explaining 47% (SE = 0.07) of the phenotypic variation (Supplementary Table 6) and 93% (SE = 0.04) of the *SNP-h*^*2*^ (Supplementary Table 7). The weakest factor loading was found for word reading speed (Fig. 1a, b), explaining only 27% (SE = 0.07) of the phenotypic variation (Supplementary Table 6), but 82% (SE = 0.09) of the *SNP-h*^*2*^ (Supplementary Table 7). Thus, the shared genetic factor captured the vast majority of genetic variance across all reading fluency measures, and its factor structure is reflected by near-perfect genetic correlations (*r*_g_) between the different reading fluency measures (Supplementary Table 8 and Fig. 1c). For example, passage reading accuracy had *r*_g_ of 0.97 (SE = 0.02) with non-word reading accuracy, 0.92 (SE = 0.04) with non-word reading speed, 0.92 (SE = 0.03) with word reading accuracy, 0.88 (SE = 0.05) with word reading speed, and 0.93 (SE = 0.04) with passage reading speed.

There was further evidence for measurement-specific factors describing additional genetic variation underlying non-word reading speed (passing the nominal *p*-value threshold only), word reading speed and accuracy, and passage reading accuracy (Fig. 1a, b), although the explained phenotypic variation was small. Word reading speed (age 13) had the strongest measurement-specific factor loading explaining 6% (SE = 0.03) of phenotypic variance (Supplementary Table 6), corresponding to 18% (SE = 0.09) of the *SNP-h*^*2*^ (Supplementary Table 7).

To ensure that the conclusion from Stage 2 analysis would be robust across the choice of the reading fluency measure, we selected two proxy measures based on the structural model for reading fluency in Stage 1: (i) passage reading accuracy (age 9), because it showed the highest common factor loading for reading, and (ii) word reading speed (age 13), because it showed the highest measurement-specific genetic factor loading.

### Single-domain structural models of spelling

As a second proxy identification analysis in Stage 1, we fitted a saturated Cholesky model to spelling accuracy scores (Supplementary Table 5) at 7 and 9 years of age (Supplementary Fig. 2 and Supplementary Tables 10–12). This model showed that genetic factors underlying spelling accuracy at age 7 years can capture nearly all (94% with SE = 0.09) of the genetic variation for spelling accuracy at age 9 years (Supplementary Table 11), with *r*_g_ of 0.97 (SE = 0.05, Supplementary Table 12). Consequently, the former measure was selected as a proxy for Stage 2 analysis.

### Multi-domain structural models

**Fig. 2:**
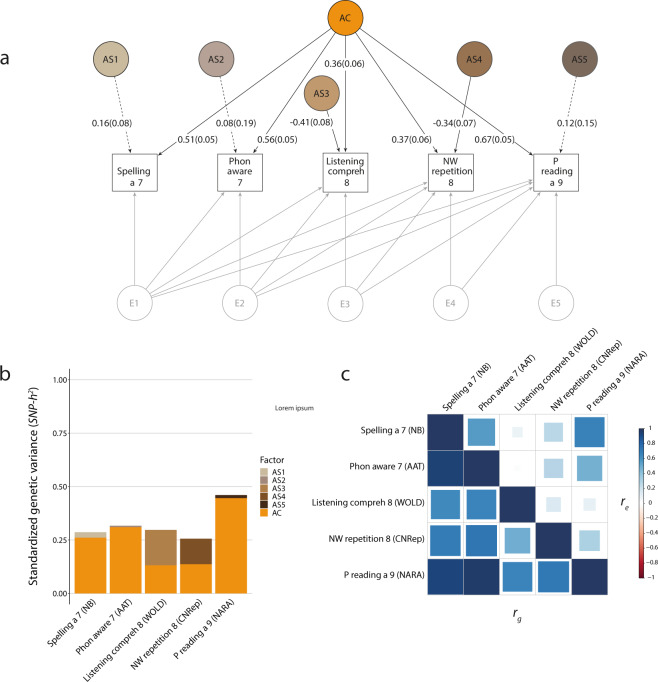
Multi-domain structural model (Passage reading subset). Genetic-relationship matrix structural equation modeling (GSEM) of literacy, phonological awareness, language and PWM abilities (*N* = 6453). The path diagram (**a**) depicts the genetic factors of the best-fitting model (IPC model) describing variation in spelling accuracy at age 7 (Spelling a 7, NB), phonemic awareness at age 7 (Phon aware 7, AAT), listening comprehension at age 8 (Listening compreh 8, WOLD), non-word repetition at age 8 (NW repetition 8, CNRep), and passage reading accuracy at age 9 (P reading a 9, NARA). The phenotypic variance was dissected into common (AC) and specific (AS1-AS5) genetic factors, according to an Independent Pathway model, as well as residual factors (E1-E5), based on a Cholesky decomposition (Factor loadings are not shown, Supplementary Table 13, Supplementary Fig. 4). Observed measures are represented by squares and factors by circles. Single-headed arrows (paths) define relationships between variables. Dotted and solid paths represent factor loadings with *p* > 0.05 and *p* ≤ 0.05 respectively. The variance of latent variables is constrained to unit variance; this is omitted from the diagram to improve clarity. The variance plot (**b**) depicts the standardized genetic variance components for the model in **a**. The correlogram (**c**) shows genetic (*r*_g_, lower triangle) and residual (*r*_e_, upper triangle) correlations for the model in **a**. Point estimates and their SEs are reported in Supplementary Table 16. All measures were rank-transformed. IPC model – Combined Independent Pathway/Cholesky model.

**Fig. 3:**
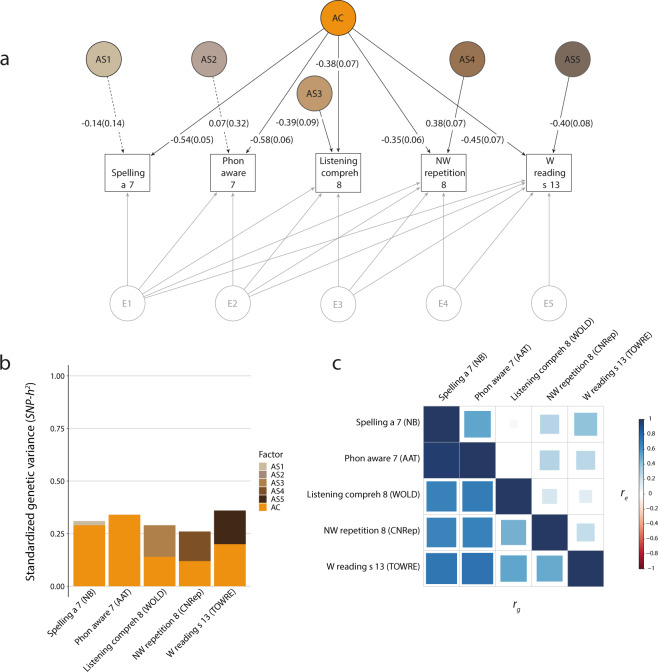
Multi-domain structural model (Word reading subset). Genetic-relationship matrix structural equation modeling (GSEM) of literacy, phonological awareness, language and PWM abilities (*N* = 6383). The path diagram (**a**) depicts the genetic factors of the best-fitting model (IPC model) describing variation in spelling accuracy at age 7 (Spelling a 7, NB), phonemic awareness at age 7 (Phon aware 7, AAT), listening comprehension at age 8 (Listening compreh 8, WOLD), non-word repetition at age 8 (NW repetition 8, CNRep), and word reading speed at age 13 (W reading s 13, TOWRE). The phenotypic variance was dissected into common (AC) and specific (AS1-AS5) genetic factors, according to an Independent Pathway model, as well as residual factors (E1-E5), based on a Cholesky decomposition (Factor loadings not shown, Supplementary Table 17, and Supplementary Fig. 4). Observed measures are represented by squares and factors by circles. Single-headed arrows (paths) define relationships between variables. Dotted and solid paths represent factor loadings with *p* > 0.05 and *p* ≤ 0.05 respectively. The variance of latent variables is constrained to unit variance; this is omitted from the diagram to improve clarity. The variance plot (**b**) depicts the standardized genetic variance components for the model in **a**. The correlogram (**c**) shows genetic (*r*_g_, lower triangle) and residual (*r*_e_, upper triangle) correlations for the model in **a**. Point estimates and their SEs are reported in Supplementary Table 20. All measures were rank-transformed. IPC model – Combined Independent Pathway/Cholesky model.

In Stage 2 analyses, we investigated the multivariate genetic covariance of literacy skills (reading fluency and spelling), phonological awareness (phonemic awareness), oral language (listening comprehension), and PWM (non-word repetition). Each of the proxy measures for reading fluency was independently studied. The multi-domain model with passage reading accuracy (age 9) as proxy for reading fluency was referred to as passage reading subset (Fig. 2), while the multi-domain model with word reading speed (age 13) as proxy was referred to as word reading subset (Fig. 3). For each analysis (Tables 3 and 4), we compared the fit of a saturated Cholesky decomposition to the fit of an independent pathway and IPC model.

**Table 3:** Multi-domain GSEM model fit comparisons of passage reading subset.

Model	LL	-2LL	*k*	AIC	BIC	Δ*χ*^2^ to Cholesky	Δ*df* to Cholesky	*p* value
Cholesky	−8909.53	17819.06	30	17879.06	18082.23	–	–	–
IPC	−8910.21	17820.41	25	17870.41	**18039.72**	1.35	5	0.93
IP	−8961.81	17923.61	20	17963.61	18099.06	104.55	10	<10^−10^

6453 participants had phenotype data of this domain and genetic data. The models were compared with likelihood ratio tests, AIC and BIC. The model with the lowest BIC values is shown in bold and its path diagram is shown in Fig. 2. All analyses are based rank-transformed residualized scores.

*AIC* Akaike information criterion, *BIC* Bayesian information criterion, *Cholesky* Cholesky decomposition (saturated) model, *GSEM* genetic-relationship matrix structural equation modeling, *IPC* joint independent pathway (genetic part)/Cholesky (residual part) model, *IP* independent pathway model, *LL* log-likelihood, *k* number of parameters.

**Table 4:** Multi-domain GSEM model fit comparisons of word reading subset.

Model	LL	-2LL	k	AIC	BIC	Δ*χ*^2^ to Cholesky	Δ*df* to Cholesky	*p* value
Cholesky	−10105.56	20211.12	30	20271.12	20473.96	–	–	–
IPC	−10107.59	20215.19	25	20265.19	**20434.22**	4.07	5	0.54
IP	−10150.18	20300.36	20	20340.36	20475.58	89.23	10	<10^−10^

6383 participants had phenotype data of this domain and genetic data. The models were compared with likelihood ratio tests, AIC and BIC. The model with the lowest BIC values is shown in bold and its path diagram is shown in Fig. 3. All analyses are based rank-transformed residualized scores.

*AIC* Akaike information criterion, *BIC* Bayesian information criterion, *Cholesky* Cholesky decomposition (saturated) model, *GSEM* genetic-relationship matrix structural equation modeling, *IPC* joint independent pathway (genetic part)/Cholesky (residual part) model, *IP* independent pathway model, *LL* log-likelihood, *k* number of parameters.

For the passage reading analysis, the IPC model fitted the data best, based on AIC, BIC, and LRTs (Table 3 and Supplementary Tables 13–16). There was evidence for a single shared genetic factor across all the abilities studied (Fig. 2a and Supplementary Table 13), capturing between 13% (SE = 0.04) and 45% (SE = 0.07) of the phenotypic variance of each trait and, thus, a large proportion of *SNP-h*^*2*^ (Fig. 2b, factorial co-heritabilities >44%; Supplementary Table 14). This is reflected in moderate to strong genetic correlations between traits (*r*_g_ = 0.49–0.97), even between passage reading accuracy and listening comprehension (*r*_g_ = 0.66 with SE = 0.11, Fig. 2c and Supplementary Table 15). The shared genetic factor explained 27% (SE = 0.05) of the phenotypic variance for spelling accuracy, 31% (SE = 0.06) for phonemic awareness and 45% (SE = 0.07) for passage reading, but only 13% (SE = 0.04) for listening comprehension and 14% (SE = 0.04) for non-word repetition (Fig. 2b and Supplementary Table 13). This corresponds to 91% (SE = 0.08), 98% (SE = 0.1), 97% (SE = 0.08), 44% (SE = 0.14), and 53% (SE = 0.14) of the genetic variance for each measure respectively (Supplementary Table 14). Thus, the majority of genetic variance across all traits (>90%) is captured by a shared genetic factor. Additional measurement-specific genetic influences, reflecting here trait-specific genetic variation, were estimated for listening comprehension and non-word repetition (Fig. 2a). These factors explained 17% (SE = 0.06) and 12% (SE = 0.05) of the phenotypic variance respectively (Supplementary Table 13 and Fig. 2b), corresponding to 56% (SE = 0.14) and 47% (SE = 0.14) of the *SNP-h*^*2*^ (Supplementary Table 14).

Identified multivariate model structures for the second dataset, including the reading fluency proxy word reading speed (age 13) (Fig. 3), assessed ~4 years later than passage reading accuracy (age 9), confirmed the findings above. Model fitting indices, again, suggested a marginally better fit of the IPC model, based on AIC, BIC and LRTs (Table 4 and Supplementary Tables 17–20). Thus, consistent genetic factor structures between reading fluency, spelling, phonemic awareness, listening comprehension and non-word repetition (Supplementary Tables 15 and 19) could be robustly identified using different multivariate structural models, irrespective of the genetic composition of the selected reading fluency measure. However, in contrast to the model for the passage reading dataset, the shared factor only captured 56% (SE = 0.14) of the *SNP-h*^*2*^ for word reading speed (age 13), while the remaining 44% (SE = 0.14) were captured by a trait-specific factor (Supplementary Table 18). This pattern mirrors the large specific factor for word reading speed estimated with the Stage 1 reading fluency model (Fig. 1a). Nonetheless, estimated genetic correlations between word reading speed and other phenotypes were still moderate to high; they were 0.52 (SE = 0.11) with listening comprehension, 0.50 (SE = 0.10) with non-word repetition, 0.72 (SE = 0.10) with spelling and 0.74 (SE = 0.10) with phonemic awareness (Fig. 3c and Supplementary Table 20).

A sensitivity analysis confirmed that the bivariate genetic correlations estimated with GSEM and GCTA, were nearly identical (Supplementary Tables 3 and 4). Furthermore, all multivariate GSEM-*SNP-h*^*2*^ (Supplementary Tables 8, 12, 15, and 19) were consistent with univariate GCTA *SNP-h*^*2*^ estimates (Supplementary Table 1), based on derived 95% confidence intervals (CIs).

Lastly, we compared multivariate genetic and residual trait correlation patterns as fitted by IPC models. Among reading fluency measures (Fig. 1c), and extending to spelling accuracy and phonemic awareness (Figs. 2c and 3c), strong positive genetic correlations (>0.7) matched modest to strong residual correlations (0.27–0.80) with the same direction of effects. Residual correlations among literacy measures, with age differences spanning up to 6 years, were still moderate. For example, residual correlations were estimated at 0.67 (SE = 0.03) and 0.39 (SE = 0.06) between spelling accuracy (age 7) and, both passage reading accuracy (age 9) (Fig. 2c and Supplementary Table 16) and word reading speed (age 13) (Fig. 3c and Supplementary Table 20) respectively. Genetic and residual correlation patterns across different domains, especially listening comprehension and literacy, were more diverse. While there was evidence for moderate to strong genetic correlations across all domains, the respective residual correlations were weak or null (zero within the 95% CIs), as shown in Supplementary Tables 16 and 20. Especially, the residual correlations between listening comprehension versus passage reading, spelling accuracy, and phonemic awareness, were low in comparison to genetic correlations (Fig. 2c); respective residual correlations were 0.08 (SE = 0.05), 0.02 (SE = 0.05) and 0.11 (SE = 0.07), while corresponding genetic correlations were 0.64 (SE = 0.11), 0.66 (SE = 0.11), and 0.66 (SE = 0.11) (Fig. 2c and Supplementary Table 16). Likewise, most of the phenotypic covariance was accounted for by genetic covariance, with bivariate *SNP-h*^*2*^ estimates consistent with one, based on derived 95% CIs (Supplementary Table 15).

## Discussion

By modeling multivariate genetic variance within a population-representative sample of unrelated youth using genome-wide markers, we demonstrated that literacy, phonological awareness, language and PWM abilities share a large proportion of their underlying genetic variation, but also revealed substantial differences in their genetic variance composition. These findings imply a pleiotropic set of common genetic variants that contributes broadly to performance in these domains and can be augmented by trait-specific genetic variation. Together with evidence for discordant genetic and residual covariance patterns, especially between genetically highly correlated oral language and literacy abilities, our findings suggest differences in underlying etiological mechanisms.

In line with long-established findings from multivariate twin research^14,18,23^, we identified an overarching genetic (core) factor that is not only shared across literacy skills, including non-word, word, passage reading abilities and spelling, but also with other abilities such as phonemic awareness, listening comprehension, and non-word repetition that are foundational for the acquisition and use of literacy skills. Utilizing passage reading accuracy (age 9) as a reading fluency proxy, the overarching genetic factor accounted almost fully (>90%) for the genetic variance in reading fluency, spelling and phonemic awareness and, to a lesser extent, for the genetic variance in listening comprehension (44%) and non-word repetition (53%). The identified shared genetic factor suggests wide-spread pleiotropy, in support of the concept of “generalist genes”^27^ that, here, may capture multiple shared aspects of cognitive functioning related to decoding abilities. The shared genetic covariance structures also include genetic overlap between listening comprehension and reading fluency, and extend findings from previous twin research^15,40^ that detect primarily moderate genetic correlations between oral language and reading fluency of 0.47–0.58 (across ages 7, 12, and 16 years^14^). The connection between oral language and reading fluency may arise due to several processes: Not all words can be acquired through phonological skills, as predicted by the “Simple View of Reading”^4,7^; words with inconsistent orthographic – phonological mappings need to be memorized or used in conjunction with contextual cues^15^. Furthermore, once decoding skills have been established and well-practiced on reading words, the orthographic representations of those words become integrated “lexical representations” directly connected to phonological and semantic knowledge^15,41^. Likewise, phonological awareness is no longer simply a predictor of reading ability, but instead becomes reciprocally shaped by reading experience^1,42^. These processes potentially strengthen the covariance between language, literacy, and phonological awareness with reading experience, as children accumulate increasingly detailed orthographic knowledge^1^.

Multivariate genetic variance models also demonstrated that non-word repetition, here considered a proxy of PWM, shares genetic variation with listening comprehension, literacy abilities, and phonemic awareness. These findings extend twin research reporting moderate genetic correlations between early-childhood non-word repetition and a mid-childhood reading composite measure (*r*_g_ = 0.44)^43^ and emphasize the importance of general cognitive resources across domains during mid-childhood. Based on the core genetic factor contribution to phenotypic variance (factorial co-heritabilities), our structural models suggest that shared etiological processes contribute to nearly half of the genetic variance for both, PWM and language. Our findings support also recent investigations of working memory, based on genome-wide genotyping information from unrelated individuals, using summary statistics^44^. This research suggested that both verbal numerical reasoning and memory-related tasks are related to an underlying common genetic factor^44^, which has long been hypothesized by observational studies^45^.

The genetic core factor across all domains studied here could be robustly identified using two diverse reading fluency proxies ascertained at different developmental stages, spanning mid-childhood to early adolescence. Longitudinal phenotypic research has demonstrated that word decoding skills are age-dependent, shaping the transition of “learning to read” to “reading to learn”^24,46^. In contrast, etiological evidence from twin studies has confirmed a high level of genetic stability for word-level decoding^14^, with age-to-age (7–12–16 years) genetic correlations of 0.72–0.84. Similarly, our findings confirm a high level of developmental stability in the genetic structure of reading fluency using very different methods.

We also observed evidence of at least three instances where genetic influences reflect specific genetic variation. First, there was a trait-specific genetic variance contribution to oral language abilities (here listening comprehension), consistent with the “Simple View of Reading”^4,7^ distinguishing language comprehension from decoding abilities. This provides converging evidence with recent twin models suggesting a strong patterning of oral language with reading comprehension, compared to a distinct and only moderate overlap between oral language with reading fluency^14^. Furthermore, word recognition (decoding) and listening comprehension have been shown to exert independent genetic influences on reading comprehension^16^, e.g. children with high reading accuracy and fluency are not necessarily efficient in reading comprehension^47^.

Second, we identified a trait-specific genetic variance contribution underlying non-word repetition. PWM is thought to consist of two components; (i) a short-term phonological store, as the non-word has to remain in memory long enough to identify the individual phonemes and their sequence, and (ii) an articulatory rehearsal process, as the non-word has to be rehearsed sufficiently rapidly and repeatedly to prevent it from decaying over time^10^. Thus, while these processes are strongly interlinked with language and literacy, supported by findings of this study and others^44^, we also find evidence for an independent genetic contribution.

Third, the structural model across all domains identified a trait-specific genetic factor for reading fluency, as proxied by word reading speed (Fig. 3), but not passage reading accuracy (Fig. 2). As genetic variance in word reading speed, too, could only partially be explained by the common genetic factor shared with other reading fluency measures (82%: Fig. 1 and Supplementary Table 6, Stage 1 analysis), these genetic contributions are likely to represent measurement-specific influences. To the best of our knowledge, there are currently no studies that have directly examined the genetic links between passage and word reading fluency. The differences in genetic structures may reflect the fact that reading accuracy and speed measures, based on grammatically and semantically coherent passages, are likely to be more influenced by comprehension skills compared to those based on single-word reading^1,48^.

Beyond genetic correlations, literacy and phonological awareness measures were, without exception, also residually intercorrelated. Residual trait covariance reflects the phenotypic trait covariance that is not captured by GRMs derived from unrelated individuals. As weak to strong residual correlations were observed across different psychological instruments and raters across a window of ~6 years, correlated assessment-specific error is an unlikely explanation. Residual correlations may also reflect the influence of rare genetic variation although this, too, is an unlikely scenario, given the low power of population-based cohorts to detect rare genetic effects^49^. Instead, residual correlations are likely to reflect here shared environmental factors such as neighborhood, but also the English schooling system. As such, our findings suggest transferability of acquired skills across literacy and phonological awareness domains. This conclusion is strongly supported by twin study findings of bi-directional effects between reading fluency and reading comprehension and overlapping genetic and environmental factors^50^. In contrast, residual correlations between oral language versus literacy or phonological awareness abilities were near zero, despite moderate genetic relationships. In conjunction with the identified trait-specific genetic variance contributions for listening comprehension, these findings provide converging evidence for differences in underlying etiological mechanisms between oral language versus literacy/phonological awareness skills. Moreover, they implicate reduced modifiability of listening comprehension by processes that shape literacy skills during mid-childhood to early adolescence. Although the nature of these etiological mechanisms is not yet known, our findings highlight the importance of studying the scope of schooling and intervention programs targeting reading and language.

This study benefits from modeling multivariate genetic trait covariance using directly assessed genomic data, and likelihood-ratio-test-based model comparison against a saturated model. GSEM^32^, thus, extends GRM-based methods, such as GREML^36^, by incorporating models that are popular in multivariate twin studies. The GSEM approach takes advantage of (i) accurate estimations of genetic relatedness between unrelated individuals using genomic data, (ii) similar recruitment conditions for all participants, including comparable environmental conditions (e.g. UK schooling system), and, consequently, and (iii) general population-based findings through use of the ALSPAC cohort^33^. A further strength of this work is the introduction of IPC models that jointly enhance both the interpretability of model coefficients and the accuracy of estimates, while incorporating information from all available data. Importantly, our analyses show that unmodeled shared residual variation can strongly affect the model fit (LRT-*p* < 10^−10^). Thus, GSEM also complements and extends existing twin research methodologies by presenting new models to study genetic trait interrelationships in samples of unrelated individuals with dense phenotyping information, such as ALSPAC.

Our results should be interpreted in light of several limitations. First, the abilities studied here were all assessed during mid-childhood and adolescence. ALSPAC lacks longitudinal information on these measures that would allow assessing measurement-invariant, development-specific genetic variance changes, such as previously reported for age-dependent word decoding skills^24,46^. However, using a latent variable approach, as presented in this study, still facilitates the identification of shared and specific genetic variance components. Second, population-level phenomena, such as assortative mating and dynastic effects, can potentially inflate genetic correlations between assessed measures and upward-bias SNP heritability, even in seemingly unrelated individuals^51^. For example, parents may select each other based on their compatibility in cognitive skills, which may create a reading-rich environment for their children. These phenomena could explain some genetic covariation across measures in our study. However, population-level biases should systematically affect all intercorrelated traits and are, thus, unlikely to account for differences in genetic and residual correlation patterns between language and literacy measures. Furthermore, this bias is not unique to GSEM or methods analyzing genetic covariance structures in unrelated individuals. Violation of the random mating assumption also introduces bias to genetic variance estimates in twin studies, although the direction of bias would be opposite; with an overestimation of the shared environmental and an underestimation of the shared genetic effect^31^. Finally, the lack of independent cohorts with comparable sets of literacy, phonological awareness, language, and PWM measures prevents a direct replication of our work, although the consistency of our findings with existing research study reports supports the validity of our results.

Modeling multivariate genome-wide genetic covariance, we have identified an overarching, pleiotropic genetic factor that is shared among measures of literacy, phonological awareness, language, and PWM. This core genetic factor is augmented by trait-specific genetic variance contributions, especially for oral language and PWM. Together with evidence for distinct genetic and residual covariance structures across oral language and literacy, our findings suggest a diverse spectrum of interrelated cognitive skills involving multiple etiological mechanisms and different levels of trait modifiability during mid-childhood and adolescence.

## Methods

### Cohort description

This study was carried out using longitudinal data from children and adolescents between 7 and 13 years from the ALSPAC study, a UK population-based pregnancy-ascertained birth cohort (estimated birth date: 1991–1992)^33,34^.

Written informed consent was obtained where appropriate for the use of data collected via questionnaires and clinics was obtained from participants following the recommendations of the ALSPAC Ethics and Law Committee at the time. Consent for biological samples has been collected in accordance with the Human Tissue Act (2004). Details on the recruitment of the ALSPAC cohort can be found in Supplementary Note 1.

Please note that the study website contains details of all the data that is available through a fully searchable data dictionary and variable search tool: http://www.bristol.ac.uk/alspac/researchers/our-data/.

### Phenotype descriptions

Traits in this study included reading fluency (non-word reading speed and accuracy, word reading speed and accuracy, passage reading speed and accuracy), spelling (accuracy), phonemic awareness, listening comprehension, and non-word repetition. These measures were ascertained using standardized as well as ALSPAC-specific instruments (Table 1). We excluded ALSPAC measures that were composites of multiple language and literacy domains (such as e.g. verbal intelligence) or which involved tiered assessments.

#### Word reading accuracy age 9 (NBO)

To evaluate word reading accuracy, the child was asked to read aloud a list of 10 real words. Nunes and Bryant selected these real words, which are a subset of those proposed by Nunes, Bryant, and Olsen (NBO)^52^. The reading accuracy score consists of the total number of items that the child read correctly. The test–retest reliability was 0.80, and the score had a correlation of 0.83 with the Schonell Word Reading Task^53^.

#### Passage reading speed and accuracy age 9 (NARA II)

Children’s passage reading speed and accuracy were assessed with the revised Neale Analysis of Reading Ability^54^ (NARA II). A story was given to the child to read. The tester recorded the time it took the child to read each passage, and noted any errors made by the child. All scores were standardized by age. Depending on age at assessment, the parallel form reliability for accuracy ranged from 0.84 to 0.92 (average 0.89), and from 0.50 to 0.83 (average 0.66) for speed^55^. Reading speed and accuracy had a correlation of 0.76 and 0.95 with the Schonell Graded Word Reading Test^53^ respectively.

#### Word reading speed age 13 (TOWRE)

The Test of Word Reading Efficiency^56^ (TOWRE) was used to assess overall word reading efficiency using the Sight Word Efficiency sub-scale. The child was given 45 seconds to read as many words as possible. Words that were skipped or wrong words were marked by the tester. The reading speed score was computed as the total number of correct words read by the child. Depending on age and sub-task, the alternate form reliability for this test ranged between 0.86 and 0.97 (with an average of 0.93), and correlations with the Woodcock Reading Mastery Tests ranged from 0.89 to 0.94, demonstrating concurrent validity^55^.

#### Non-word reading accuracy age 9 (NBO)

The child was asked to read aloud 10 non-words. These words had been selected from a larger selection of non-words using an ALSPAC-specific instrument based on the research conducted by Nunes and colleagues^52^. The test–retest reliability of the non-word reading task was 0.73 and the score had a correlation of 0.73 with the Schonell Word Reading Task^53^. The tester emphasized to the child that the words were made-up and asked the child to read all the non-words in the way that they thought they should be read. A total score was computed as the sum of the number of items read correctly by the child based on regular symbol-sound correspondences of written English.

#### Non-word reading speed age 13 (TOWRE)

The Test of Word Reading Efficiency (TOWRE) was also used to assess non-word reading speed using the Phonemic Decoding Efficiency sub-scale. It is designed as a relatively pure measure of decoding, independent of meaning. The child had 45 seconds to read as many non-words as possible. Non-words that a child skipped or got wrong were marked by the tester. A total score was computed as the sum of the number of correct non-words read by the child based on regular symbol-sound correspondences of written English. The alternate form reliability was 0.94 and the test–retest reliability ranged between 0.82 and 0.97^55^. For validity, TOWRE had correlations with the Woodcock Reading Mastery Tests ranged between 0.89 and 0.91^55^.

#### Spelling accuracy age 7 and age 9 (NB)

Spelling accuracy was assessed by asking a child to spell a series of 15 words. The words were chosen specifically for this age group after piloting on several hundred children (Nunes and Bryant, ALSPAC-specific measure^23^). The words included regular and irregular words of differing frequencies and were put in an order of increasing difficulty. For each word, the tester first read the word out alone to the child, then within a specific sentence incorporating the word, and finally alone again. The child was then asked to write down the spelling. The spelling accuracy score is computed as the number of words spelt correctly. The test–retest reliability was >0.7 and scores were 0.91 correlated with the Schonell Spelling Test^52^.

#### Phonemic awareness age 7 (AAT)

Phonemic awareness was assessed using the Auditory Analysis Test^57^ (AAT). The task contained two practice and 40 test items of increasing difficulty. For each item, the child was first asked to repeat the word, and then to produce it again but with part of the word (a phoneme or several phonemes) removed. For example, the word to repeat initially is “sour” and then the child is asked to repeat it again without /s/ to which the correct response is “our”. The test assessed seven omission categories: the omission of a first, medial or final syllable, the omission of the initial consonant, the omission of the final consonant of a one-syllable word, and the omission of the first consonant or consonant blend of a medial consonant. The words from different categories were mixed. Reliability of the AAT was not assessed in the initial task report^57^. However, test criteria of the commercial version of the AAT^58^ have been assessed and revealed high internal consistency (0.78). A total score was computed as the sum of correct responses over all types of omission. The AAT had a correlation of 0.53–0.84 with the language arts subsets of the Stanford Achievement Test^57^.

#### Listening comprehension at 8 (WOLD)

Listening comprehension is a subtest of the Wechsler Objective Language Dimensions (WOLD)^59^. A picture was shown to the participant, and the examiner read aloud a paragraph about the picture. The participant then answered multiple open-ended questions on what they have heard. The listening comprehension subtest has test-retest reliabilities between 0.83 and 0.88 in children aged 6–11 years^60^ and the correlation with the Peabody Picture Vocabulary Test-III^61^ was 0.44^62^.

#### Non-word repetition at age 8 (NWR)

Children’s short-term memory was evaluated by a non-word repetition test^11^. The test contains 12 nonsense words, for each of 3, 4, and 5 syllables, all conforming to English rules for sound combinations. The participant was asked to listen to each of these 36 words from an audio cassette recorder and then repeat each word. The test–retest reliability was 0.8 and NWR scores showed correlations between 0.45 and 0.67 with the digit span test^11^.

### Phenotype transformations

Any children who stopped prematurely during the psychological tests were excluded from the study. The scores of each test were adjusted for age, sex, and the two principal components from the genotyping analysis to correct for population stratification^63^. The scores from passage reading measures (NARA) are already age-standardized, and therefore these scores were not further adjusted for age. All residualized scores were finally rank-transformed to improve the GSEM log-likelihood estimation assuming multivariate normality.

*SNP-h*^*2*^ estimates (Supplementary Table 1) and bivariate phenotypic correlations (based on Pearson correlation coefficients, Supplementary Table 2) between measures, using both untransformed and rank-transformed scores, were comparable with each other and with previous reports^23^.

### Genotyping

ALSPAC children were genotyped using Illumina HumanHap550 quad-chip platform. The ALSPAC GWAS data were generated by Sample Logistics and Genotyping Facilities at the Wellcome Trust Sanger Institute and LabCorp (Laboratory Corporation of America) using support from 23andMe. After quality control (individual call rate > 0.97, SNP call rate > 0.95, Minor allele frequency (MAF) > 0.01, Hardy-Weinberg equilibrium (HWE) *p* > 10^−7^, and removal of individuals with cryptic relatedness and non-European ancestry), genome-wide data were available for 8226 children and 465,740 directly genotyped single-nucleotide polymorphisms (SNPs). Among those children, 6774 had at least one phenotype observation for reading fluency, spelling, phonological awareness, language, or PWM abilities available.

### Genetic-relationship matrix structural equation modeling

Multivariate genetic covariance structures were identified using genetic-relationship matrix structural equation modeling (R:gsem library, version 1.1.0, https://gitlab.gwdg.de/beate.stpourcain/gsem)^32^. This multivariate analysis technique combines whole-genome genotyping information with structural equation modeling techniques applied in twin research to model multivariate genetic variance using a maximum likelihood approach^32^. Thus, within the GSEM framework, genetic and residual variance were modeled as genetic and residual factors, respectively. More specifically, GSEM also allows modeling multivariate residual influences, such as those not captured by genotyped markers, potentially involving rare, non-additive or un-tagged genetic influences, environmental risk factors, and random error^36^. In this study, phenotypic variance for each measure was dissected into genetic and residual influences (AE model) using a full Cholesky decomposition and an independent pathway model^32,35^:

i. The Cholesky decomposition model is a fully parametrized descriptive model without any restrictions on the structure of latent genetic and residual influences. This saturated model can be fit to the data through decomposition of both the genetic variance and residual variance into as many genetic and residual factors as there are observed variables.
ii. The independent pathway model specifies a common genetic and a common residual factor, in addition to trait-specific genetic and residual influences.
iii. The combined Cholesky decomposition and independent pathway (IPC) model structures the genetic variance as an independent pathway model (consisting of common and measurement-specific influences) and the residual variance as a Cholesky decomposition model (where the number of residual factors is the same as the number of observed variables). Supplementary Figure 3 shows an example of an IPC model with six traits, and Supplementary Fig. 4 an example of an IPC model with five traits.

The goodness-of-fit of GSEM models to the data was evaluated with LRTs, AIC and BIC^39^.

In the present study, we modeled rank-transformed residualized phenotypes (adjusted for age, sex, and the first two principal components) as described above. Genetic-relationship matrices were constructed based on directly genotyped variants in unrelated individuals, using GCTA software^36^. Individuals with a relatedness >0.05 (off-diagonals within a genetic-relatedness matrix) were excluded. Factor loadings were evaluated using Wald tests.

Multivariate models in unrelated individuals, studying interindividual genetic variation captured by genome-wide genetic variation, are computationally expensive^32^. For example, a 6-factor Cholesky decomposition model, as fitted within this study, can require 6 weeks computing time even on a system incorporating at least 4 parallel cores of 3 GHz and between 50 and 75 Gb memory. Hence, we started the modeling process by strategically combining similar measures, such as reading fluency or spelling abilities, to reduce the number of studied instruments by selecting proxies that most comprehensively capture the shared genetic variance of an entire domain. The advantage of this approach is that we can control the extent to which selected proxies represent underlying shared genetic factors, either fully or along with specific measurement-related influences, and subsequently assess the stability of the identified genetic structures. The identified proxy measures in Stage 1 were eventually jointly modeled with other measures as part of the Stage 2 analysis, with models across four phenotypic domains: literacy measures, including both reading (non-word, word, and passage reading speed and accuracy) and spelling, phonological awareness (phonemic awareness), oral language (listening comprehension), and PWM (non-word-repetition).

Applying a conservative approach, we also evaluated all derived factor loadings of the Stage 2 model against an experiment-wide error rate of 0.007, estimated based on the effective number of individually analyzed phenotypes using Matrix Spectral Decomposition (MatSpD)^64^, accounting for all previously conducted statistical assessments. However, a correction for multiple testing is not directly applicable, as we jointly analyze genetic trait covariance using GSEM.

Identified structural models were used to estimate *SNP-h*^*2*^ as well as genetic correlations, factorial co-heritabilities (the proportion of total trait genetic variance explained by a specific genetic factor), and bivariate heritabilities (the contribution of genetic covariance to the observed phenotypic covariance between two measures), as defined here:

Bivariate genetic correlation between phenotypes, measuring the extent to which two phenotypes 1 and 2 share genetic factors (ranging from −1 to 1), can be derived using estimated genetic variance and covariance^65^ according to:

1 $$r_{\mathrm{g}} = \frac{{\sigma _{{\mathrm{g}}12}}}{{\sqrt {\sigma _{{\mathrm{g}}1}^2\sigma _{{\mathrm{g}}2}^2} }}$$

where $\sigma _{{\mathrm{g}}12}$ is the genetic covariance between phenotypes 1 and 2, and, $\sigma _{{\mathrm{g}}1}^2$ and $\sigma _{{\mathrm{g}}2}^2$ are the respective genetic variances.

A measure of factorial co-heritability was introduced to assess the relative contribution of a genetic factor to the genetic variance of a phenotype, estimated with the gsem package (R:gsem library, version 1.1.0). The factorial co-heritability *f*_g_^*2*^ is defined as:

2 $$f_{\mathrm{g}}^2 = \frac{{\sigma _{{\mathrm{g}}\_it}^2}}{{{\sum} {\sigma _{{\mathrm{g}}\_it}^2} }} = \frac{{\sigma _{{\mathrm{g}}\_it}^2}}{{\sigma _{{\mathrm{g}}\_t}^2}}$$

where $\sigma _{{\mathrm{g}}\_it}^2$ is the genetic variance of the genetic factor *i* contributing to trait *t* and $\sigma _{{\mathrm{g}}\_t}^2$ the total genetic variance of trait *t*, based on standardized factor loadings. The corresponding SEs were derived using the Delta method.

Bivariate heritability also known as co-heritability^66^ was defined as the ratio of the genetic to the phenotypic covariance between two traits and estimated using the gsem package (R:gsem library, version 1.1.0).

3 $$h_{{\mathrm{g}}\_biv}^2 = \frac{{\sigma _{{\mathrm{g}}12}}}{{\sigma _{{\mathrm{p}}12}}}$$

where $\sigma _{{\mathrm{g}}12}$ is the genetic covariance, estimated based on unstandardized factor loadings, and $\sigma _{{\mathrm{p}}12}$ the phenotypic covariance from observed rank-transformed measures. The respective SEs were approximated by the SE of the genetic covariance divided by the phenotypic covariance (as the SE of the phenotypic covariance is very small).

### Genetic-relationship-matrix residual maximum likelihood

The GCTA software package (v1.25.2)^36^ can be used to estimate the proportion of phenotypic variation that is explained by markers on genotyping chip arrays using GREML^36^ (AE model). Likewise, bivariate GREML^37^ can be applied to estimate genetic covariance and genetic correlations between two phenotypes.

Univariate and bivariate GREML were carried out as part of sensitivity analyses.

### Reporting summary

Further information on research design is available in the Nature Research Reporting Summary linked to this article.

## Supplementary information

Supplementary Information Multivariate Genome-wide Covariance Analyses of Literacy, Language and Working Memory Skills Reveal Distinct Etiologies

Reporting Summary

## Data Availability

The data used are available through a fully searchable data dictionary (http://www.bristol.ac.uk/alspac/researchers/our-data/). Access to ALSPAC data can be obtained as described within the ALSPAC data access policy (http://www.bristol.ac.uk/alspac/researchers/access/).

## Code availability

All analyses were performed using freely accessible software (https://gitlab.gwdg.de/beate.stpourcain/gsem). Requests for scripts or other analysis details can be sent via email to the corresponding authors.
