# Supplementary material for: Multivariate genome-wide covariance analyses of literacy, language and working memory skills reveal distinct etiologies
Source: NPJ Sci Learn. 2021 Aug 19;6:23. doi: 10.1038/s41539-021-00101-y (PMC8377061; doi:10.1038/s41539-021-00101-y)
Supplement: Supplementary file 2 — Reporting Summary [file 41539_2021_101_MOESM2_ESM.pdf]

nature portfolio

Corresponding author(s): Chin Yang Shapland and Beate St. Pourcain

Last updated by author(s): Jun 18, 2021

Reporting Summary

Nature Portfolio wishes to improve the reproducibility of the work that we publish. This form provides structure for consistency and transparency in reporting. For further information on Nature Portfolio policies, see our Editorial Policies and the Editorial Policy Checklist.

Statistics

For all statistical analyses, confirm that the following items are present in the figure legend, table legend, main text, or Methods section.

n/a	Confirmed	
☐	☒	The exact sample size (*n*) for each experimental group/condition, given as a discrete number and unit of measurement
☐	☒	A statement on whether measurements were taken from distinct samples or whether the same sample was measured repeatedly
☐	☒	The statistical test(s) used AND whether they are one- or two-sided<br>*Only common tests should be described solely by name; describe more complex techniques in the Methods section.*
☐	☒	A description of all covariates tested
☐	☒	A description of any assumptions or corrections, such as tests of normality and adjustment for multiple comparisons
☐	☒	A full description of the statistical parameters including central tendency (e.g. means) or other basic estimates (e.g. regression coefficient) AND variation (e.g. standard deviation) or associated estimates of uncertainty (e.g. confidence intervals)
☐	☒	For null hypothesis testing, the test statistic (e.g. *F*, *t*, *r*) with confidence intervals, effect sizes, degrees of freedom and *P* value noted<br>*Give P values as exact values whenever suitable.*
☒	☐	For Bayesian analysis, information on the choice of priors and Markov chain Monte Carlo settings
☒	☐	For hierarchical and complex designs, identification of the appropriate level for tests and full reporting of outcomes
☐	☒	Estimates of effect sizes (e.g. Cohen's *d*, Pearson's *r*), indicating how they were calculated

Our web collection on statistics for biologists contains articles on many of the points above.

Software and code

Policy information about availability of computer code

Data collection	No bespoke data collection software was used - ALSPAC data used is publicly available.
Data analysis	All analyses were performed using freely accessible software. Software used for quality control and analyses are detailed in the Methods section.

For manuscripts utilizing custom algorithms or software that are central to the research but not yet described in published literature, software must be made available to editors and reviewers. We strongly encourage code deposition in a community repository (e.g. GitHub). See the Nature Portfolio guidelines for submitting code & software for further information.

Data

Policy information about availability of data

All manuscripts must include a data availability statement. This statement should provide the following information, where applicable:

- Accession codes, unique identifiers, or web links for publicly available datasets
- A description of any restrictions on data availability
- For clinical datasets or third party data, please ensure that the statement adheres to our policy

The data used are available through a fully searchable data dictionary (http://www.bristol.ac.uk/alspac/researchers/our-data/). Access to ALSPAC data can be obtained as described within the ALSPAC data access policy (http://www.bristol.ac.uk/alspac/researchers/access/). Requests for scripts or other analysis details can be sent via email to the corresponding authors.

Field-specific reporting

Please select the one below that is the best fit for your research. If you are not sure, read the appropriate sections before making your selection.

☒ Life sciences ☐ Behavioural & social sciences ☐ Ecological, evolutionary & environmental sciences

For a reference copy of the document with all sections, see nature.com/documents/nr-reporting-summary-flat.pdf

Life sciences study design

All studies must disclose on these points even when the disclosure is negative.

Sample size	We used all available data from ALSPAC on literacy-, language- and PWM-related measures passing our inclusion criteria. We calculated an experiment-wide error rate for the factor loadings to keep the Type I error rate at 5%. However, using a multivariate SEM approach, this multiple testing correction is highly conservative. Details are described in full in the Methods section.
Data exclusions	Any children who stopped prematurely during the psychological tests and related children were excluded from the analysis. We excluded ALSPAC measures which were composites of multiple language and literacy domains (such as e.g. verbal intelligence) or which involved tiered assessments.These are described in full in Methods section.
Replication	Although the lack of independent cohorts with comparable literacy-, language- and PWM-related measures prevents a direct replication of our work, the consistency of our findings with many recent twin study reports supports the validity of our results. We confirmed the identified multivariate genetic architectures using reading fluency measures at different developmental stages.
Randomization	NA - not an intervention study
Blinding	NA - not an intervention study

Reporting for specific materials, systems and methods

We require information from authors about some types of materials, experimental systems and methods used in many studies. Here, indicate whether each material, system or method listed is relevant to your study. If you are not sure if a list item applies to your research, read the appropriate section before selecting a response.

Materials & experimental systems

n/a	Involved in the study	
☒	☐	Antibodies
☒	☐	Eukaryotic cell lines
☒	☐	Palaeontology and archaeology
☒	☐	Animals and other organisms
☐	☒	Human research participants
☒	☐	Clinical data
☒	☐	Dual use research of concern

Methods

n/a	Involved in the study	
☒	☐	ChIP-seq
☒	☐	Flow cytometry
☒	☐	MRI-based neuroimaging

Human research participants

Policy information about studies involving human research participants

Population characteristics	This study was carried out using longitudinal data from children and adolescents between 7 and 13 years from the ALSPAC study, a UK population-based pregnancy-ascertained birth-cohort.
Recruitment	Pregnant women resident in Avon, UK with expected dates of delivery 1st April 1991 to 31st December 1992 were invited to take part in the study. Full details can be found in Supplementary Note 1.
Ethics oversight	Written informed consent was obtained where appropriate for the use of data collected via questionnaires and clinics was obtained from participants following the recommendations of the ALSPAC Ethics and Law Committee at the time. Consent for biological samples has been collected in accordance with the Human Tissue Act (2004).

Note that full information on the approval of the study protocol must also be provided in the manuscript.
